# Mitochondrial biology and immune crosstalk in breast cancer: therapeutic opportunities and challenges

**DOI:** 10.3389/fimmu.2026.1715998

**Published:** 2026-04-22

**Authors:** Yulin Zeng, Yingjie Guo

**Affiliations:** 1 School of Basic Medical Sciences, Lanzhou University, Lanzhou, Gansu, China; 2 Arc Institute, Palo Alto, CA, United States

**Keywords:** breast cancer, immune crosstalk, immune evasion, mitochondria, tumor microenvironment

## Abstract

Mitochondria are central regulators of breast cancer progression and therapy response, acting beyond energy metabolism to integrate redox balance, regulated cell death, metabolic plasticity, and immune signaling. This review summarizes how mitochondrial metabolism, dynamics, stress signaling, and quality-control pathways shape tumor heterogeneity, immune evasion, and treatment outcomes across tumor, immune, and stromal compartments. In breast cancer, subtype-specific mitochondrial programs influence oxidative phosphorylation, fatty acid oxidation, glutaminolysis, lactate accumulation, and mtDAMP signaling, thereby contributing to immune suppression and therapeutic resistance. We further discuss how mitochondrial regulation of apoptosis, ferroptosis, cuproptosis, and pyroptosis reveals both therapeutic opportunities and unresolved limitations. Although mitochondria-targeted strategies show translational promise, their clinical application remains constrained by metabolic heterogeneity, adaptive rewiring, immune-cell liability, and insufficient biomarkers. Overall, mitochondria represent a context-dependent therapeutic axis in breast cancer.

## 1 Introduction

Breast cancer remains a leading cause of cancer-related mortality worldwide, and its management is hindered not only by genomic heterogeneity but also by profound metabolic and immunological diversity.

Mounting evidence indicates that mitochondria function far beyond ATP production, acting as dynamic hubs that integrate bioenergetics, biosynthesis, cell death regulation, and immune signaling. In breast cancer, mitochondrial remodeling—encompassing oxidative phosphorylation (OXPHOS) reprogramming, reactive oxygen species (ROS) flux, mitochondrial DNA (mtDNA) release, and quality control pathways—directly shapes both tumor cell fitness and immune cell function within the tumor microenvironment (TME).

In this review, we examine mitochondria through a dual-focus lens: as metabolic engines that sustain tumor progression and as immunomodulatory platforms that influence antitumor or protumor immunity. By integrating recent advances in mitochondrial metabolism, dynamics, stress signaling, and quality control with immune evasion and resistance mechanisms, we outline a framework linking mitochondrial biology to drug response, immune checkpoint efficacy, and therapeutic vulnerability in breast cancer. This perspective highlights mitochondria as an important interface between cancer metabolism and immunology, while also emphasizing that their therapeutic relevance remains context dependent and constrained by heterogeneity, toxicity, and immune-cell liability.

## 2 Mitochondrial metabolic programs and immune consequences in breast cancer

### 2.1 Subtype-specific metabolic heterogeneity and immune relevance

Breast cancer is the most common malignancy in women and remains a leading cause of cancer mortality worldwide (1). Clinically and molecularly, it is classified into Luminal A, Luminal B, HER2-enriched, and triple-negative breast cancer (TNBC) subtypes, each differing in receptor status, proliferation rate, and therapy response (2, 3).

Mitochondrial features are highly heterogeneous across breast cancer subtypes (4). Triple-negative breast cancer is frequently characterized by high glycolytic activity, but many TNBC models also retain substantial OXPHOS capacity and metabolic plasticity, which may create therapeutic vulnerability to mitochondrial inhibition in aggressive or therapy-resistant settings (5–10). By contrast, luminal tumors exhibit distinct mitochondrial bioenergetic states and may show relatively lower respiratory dependence in some contexts, although metabolic phenotypes remain model- and state-dependent (11, 12). HER2-enriched tumors appear metabolically intermediate and are often associated with anabolic lipid and glutamine-linked metabolic programs (13–15). Thus, rather than assigning a single dominant metabolic state to each subtype, it is more accurate to emphasize that TNBC often combines elevated glycolysis with retained or inducible OXPHOS, whereas luminal tumors also display heterogeneous mitochondrial programs depending on biological context (6, 11, 16).

Breast cancer subtypes not only differ in their reliance on glycolysis and OXPHOS, and these metabolic programs may also shape the immune microenvironment in distinct ways. In particular, high-glycolytic TNBC is closely associated with lactate accumulation, myeloid immunosuppression, lower levels of B-cell and anti-tumor immune cell infiltration, and reduced cytotoxic T-cell activity (17–19). In contrast, luminal tumors more frequently exhibit oxidative phosphorylation-driven metabolism; the immunological consequences of this are relatively less well defined, but may be relevant to treatment (20, 21). HER2-enriched tumors appear metabolically intermediate and may couple mitochondrial respiration to anabolic lipid and glutamine pathways; however, their direct immunological consequences remain less clearly defined than those of TNBC.

### 2.2 Metabolite competition and immunosuppressive signaling

In addition to OXPHOS itself, various mitochondrial pathways also contribute to the progression of breast cancer and influence immune processes. The tricarboxylic acid (TCA) cycle intermediates, such as succinate and fumarate, act as both metabolic fuels and oncometabolites. In breast cancer, inhibition of succinate dehydrogenase (SDH) has been associated with macrophage-driven tumorigenesis and immunosuppressive remodeling (22). In addition, adenylosuccinate lyase (ADSL)-generated fumarate has recently been shown to inhibit STING signaling and promote tumor immune evasion in breast cancer (23), although this remains a relatively new mechanistic insight requiring broader validation.

Glutaminolysis plays a pivotal role in supporting mitochondrial bioenergetics and redox balance, especially in aggressive breast cancer subtypes. Beyond tumor-intrinsic metabolism, however, glutamine dependence may also contribute to immunometabolic competition, as tumor-selective inhibition of glutamine metabolism has been shown to improve antitumor T-lymphocyte activity in TNBC (24). Likewise, fatty acid oxidation (FAO) not only provides ATP and NADPH under nutrient deprivation or treatment-induced stress, but may also shape the immune microenvironment. In HER2-driven breast cancer, FAO inhibition promotes a more antitumor immune milieu and enhances response to HER2-targeted therapy, while emerging evidence suggests that FAO rewiring can impair CD8+ T-cell function in metastatic settings (25, 26). Together, these findings indicate that metabolic pathways such as glutaminolysis and FAO contribute to breast cancer progression not only by sustaining tumor plasticity, but also by influencing immune suppression and therapeutic responsiveness.

### 2.3 Translational implications and current limitations

These subtype- and pathway-specific metabolic dependencies make mitochondrial metabolism an attractive therapeutic target in breast cancer. In principle, targeting OXPHOS, glutaminolysis, fatty acid oxidation, or redox regulation may reduce tumor adaptability and enhance sensitivity to chemotherapy, targeted therapy, or immunotherapy. However, the clinical translation of these strategies remains challenging because breast cancer exhibits marked metabolic heterogeneity across and within subtypes, and tumor cells can compensate by switching between glycolysis, mitochondrial respiration, and lipid metabolism under therapeutic pressure. In addition, because many of these pathways are also required for immune-cell fitness, systemic metabolic targeting may inadvertently impair antitumor immunity. Therefore, effective mitochondria-directed therapy in breast cancer will likely require precise metabolic stratification, rational combination strategies, and careful consideration of immune consequences.

## 3 Mitochondrial dynamics, stress adaptation, and immune remodeling

### 3.1 Tumor-cell mitochondrial dynamics and adaptive remodeling

Mitochondrial dynamics, encompassing continuous cycles of fission and fusion, are essential for maintaining mitochondrial function, bioenergetic balance, and cellular homeostasis (27, 28). These dynamic processes are tightly regulated by key GTPases: fusion is mediated by mitofusin 1/2 (MFN1/2) and OPA1, while fission depends on dynamin-related protein 1 (DRP1) and FIS1 (29, 30). Disruptions in mitochondrial dynamics have been increasingly recognized in cancer, with breast cancer cells often exhibiting heightened mitochondrial fission to support rapid proliferation, invasion, and survival under metabolic stress. In breast cancer, altered DRP1-dependent mitochondrial dynamics have been linked to migration, cytoskeletal remodeling, and metastatic behavior (31, 32). But recent TNBC studies suggest that excessive fission may, under some conditions, restrain metastatic signaling, underscoring the context-dependent nature of mitochondrial dynamics (33). In hormone receptor-positive (ER^+^) subtypes, estrogen signaling can modulate mitochondrial dynamics toward a more fusion-oriented state through upregulation of MFN1/2 and reduced FIS1 expression, thereby influencing mitochondrial biogenesis and metabolic profiles (34).

At present, direct breast-cancer-specific evidence linking tumor-cell mitochondrial dynamics to defined immune phenotypes remains limited. Nevertheless, these tumor-intrinsic changes are likely to influence the immune microenvironment (35, 36). Dynamic remodeling of tumor-cell mitochondria can alter reactive ROS production and reshape metabolic and stress conditions within the tumor microenvironment, thereby affecting the context in which infiltrating immune cells function (33). Thus, in breast cancer, mitochondrial dynamics should be considered not only regulators of tumor-cell adaptation, but also potential contributors to immune remodeling in the tumor microenvironment.

### 3.2 Immune and stromal consequences of mitochondrial dynamics

Beyond tumor cells, mitochondrial dynamics in immune and stromal cells also influence breast cancer progression (35). Importantly, the consequences of mitochondrial remodeling are highly cell-type specific.

In tumor-associated macrophages (TAMs), mitochondrial dynamics can influence macrophage polarization and immune function in breast cancer (37). In TNBC, targeted regulation of TAM mitochondrial dynamics has been shown to reverse the M2-like phenotype and enhance antitumor immune responses, supporting a cell-type-specific role of mitochondrial remodeling in shaping the tumor microenvironment (38). By contrast, foundational mechanistic studies have shown that disrupted mitochondrial dynamics in T cells can impair mitochondrial fitness, reduce persistence, and promote functional exhaustion (39). In breast cancer, particularly in TNBC, this concept is currently supported mainly by bioinformatic and single-cell analyses of clinical data, which have linked T-cell mitochondrial dysfunction to exhausted CD8+ T-cell states, rather than by direct animal-model evidence (40).

Mitochondrial dynamics in stromal compartments may also influence immune behavior in breast cancer, although direct evidence for classical fission–fusion dynamics in CAFs remains limited. Current breast-cancer studies more strongly support stromal mitochondrial involvement through metabolic coupling and mitochondrial transfer, whereas broader stromal populations such as cancer-associated adipocytes (CAAs) have been shown to disrupt CD8+ T-cell mitochondrial homeostasis and dynamics via lipolytic products, thereby promoting functional exhaustion and weakening the response to anti-PD-1 therapy (41). Collectively, these observations indicate that mitochondrial dynamics influence the breast cancer microenvironment through coordinated but cell-specific effects on immune activation, stromal support, and tumor adaptation.

### 3.3 Mitochondrial quality control and immune modulation in breast cancer

Mitochondrial behaviors—including fission, fusion, mitophagy, mitochondrial biogenesis, and quality control mechanisms such as the mitochondrial unfolded protein response (UPRmt) and mitochondrial-derived vesicles (MDVs)—play pivotal roles in regulating tumor immunity, although the strength of evidence differs across pathways (42, 43).

Among these, mitophagy has the clearest breast-cancer-specific relevance: recent studies show that the ATAD3A–PINK1 axis can redirect PD-L1 to mitochondria for mitophagy-dependent degradation, thereby influencing chemoimmunotherapy responsiveness, while MUC1-driven PINK1-dependent mitophagy has also been linked to breast-cancer progression (44, 45). In parallel, mitochondrial biogenesis, mediated in part by PGC-1α, contributes to breast-cancer metabolic adaptation and metastasis, and in TNBC may be reinforced by CAF-derived PGC-1α/ERRα signaling, although the well-established role of PGC-1α in supporting T-cell persistence and memory formation is based mainly on broader tumor-immunity studies rather than breast-cancer-specific models (46, 47). In contrast, breast-cancer-specific evidence directly linking UPRmt or mitochondria-derived vesicles (MDVs) to antitumor immunity remains more limited (48). Finally, mtDNA release and cGAS–STING activation are increasingly recognized as important components of breast-cancer innate immune signaling, with the potential to promote type I interferon responses and enhance tumor immunogenicity, although the magnitude and direction of this effect remain context dependent across breast-cancer subtypes (49, 50).

## 4 Mitochondria-regulated cell death: immune relevance, resistance, and therapeutic implications

### 4.1 Apoptosis and mitochondrial immune signaling in breast cancer

Mitochondria regulate various forms of regulated cell death (RCD), with apoptosis being the most studied pathway, tightly linked to mitochondrial function (51). In intrinsic apoptosis, mitochondrial outer membrane permeabilization (MOMP) is triggered by DNA damage or oxidative stress, leading to the release of cytochrome c, which activates caspases to initiate cell death (52, 53). This process is controlled by BCL-2 family proteins, with pro-apoptotic BAX and BAK promoting MOMP, while anti-apoptotic BCL-2 and MCL-1 stabilize mitochondrial integrity, preventing cell death (52, 54, 55).

In breast cancer, particularly in chemoresistant subtypes, upregulation of anti-apoptotic proteins such as BCL-2 and MCL-1 preserves mitochondrial integrity and suppresses mitochondrial apoptosis, thereby promoting tumor cell survival and therapeutic resistance (56–58). In hereditary breast cancers, BRCA1/2 mutations initially increase sensitivity to DNA-damaging agents by impairing DNA repair (59, 60). However, acquired resistance can emerge, and anti-apoptotic rewiring may contribute to this process by limiting mitochondrial apoptosis (61–63). BRCA1 itself has also been implicated in the regulation of mitochondrial apoptosis following DNA damage (64–66).

BCL-2 overexpression, particularly in ER-positive/luminal breast cancer, has long supported interest in mitochondrial apoptosis as a therapeutic vulnerability (67, 68). However, the negative overall result of VERONICA indicates that BCL-2 inhibition is unlikely to be broadly effective in unselected endocrine-resistant, post-CDK4/6 ER-positive breast cancer (53, 69). Instead, this trial highlights the biological heterogeneity of anti-apoptotic dependencies in this setting, where adaptive rewiring toward BCL-XL- or MCL-1-mediated survival may limit the activity of venetoclax (69). These findings are also relevant to future immune-targeted strategies, as hormone receptor-positive (HR+)/HER2− breast cancers are often immunologically “cold” and show limited benefit from immune checkpoint inhibitors (70, 71). Accordingly, BH3 mimetics are unlikely to substantially enhance immunotherapy unless applied in biomarker-enriched subsets with demonstrable BCL-2 dependence and a microenvironment permissive to immune reactivation (69, 71). Preclinical studies suggest that venetoclax can reshape intratumoral immune composition and augment antitumor responses in combination with checkpoint blockade, but this concept remains clinically unvalidated in breast cancer (72, 73).

In parallel, modulation of the mitochondrial permeability transition pore (MPTP) remains mechanistically attractive as another means of promoting apoptosis, although its therapeutic window in solid tumors is still uncertain (74, 75).

Overall, mitochondrial apoptosis-related pathways, including BCL-2, MCL-1, and MPTP-associated signaling, remain of therapeutic interest, but their clinical value in breast cancer will depend on better biomarker selection, management of adaptive anti-apoptotic rewiring, and minimization of on-target toxicity.

### 4.2 Ferroptosis, cuproptosis, and pyroptosis in breast cancer immunity

In breast cancer, ferroptosis is of particular interest in metabolically stressed and therapy-resistant subtypes, especially TNBC (76, 77). Mitochondria contribute to ferroptotic susceptibility through TCA-cycle activity, ROS generation, glutaminolysis, and iron-dependent lipid peroxidation (78, 79). However, breast cancer evidence remains strongest at the level of tumor-cell metabolism and subtype-specific vulnerability, whereas its direct immunological significance is less clearly defined (77, 80). Although ferroptosis may interact with immune phenotypes and expose actionable vulnerabilities in aggressive disease, direct evidence linking ferroptosis to durable antitumor immune remodeling in breast cancer remains limited (81, 82).

By contrast, the role of cuproptosis in breast cancer is still largely exploratory (83, 84). Its dependence on mitochondrial respiration and lipoylated TCA-cycle proteins makes it mechanistically attractive, particularly in OXPHOS-dependent tumors (85, 86). Nevertheless, current breast cancer evidence is derived mainly from emerging mechanistic studies, bioinformatic analyses, and pan-cancer inference rather than direct functional validation (83, 87, 88). As such, the relevant breast cancer contexts, immune consequences, and therapeutic selectivity of copper-targeted interventions remain uncertain (89–91).

Pyroptosis has a clearer conceptual link to immune signaling, as gasdermin-mediated membrane rupture releases inflammatory mediators and mitochondrial ROS or mtDNA may promote inflammasome activation (92–94). In breast cancer, this places pyroptosis at the interface of tumor cell death and microenvironmental reprogramming (95, 96). However, most supporting evidence remains preclinical, and its dual role must be acknowledged: pyroptosis may enhance antitumor immunity in some contexts but also sustain tumor-promoting inflammation in others (97). Therefore, despite its immunological relevance, its therapeutic exploitation in breast cancer remains context dependent (95, 98).

### 4.3 Therapeutic implications and unresolved questions

Overall, these mitochondrial-linked death pathways represent conceptually important but unequally developed therapeutic opportunities in breast cancer. Among them, ferroptosis has the strongest breast cancer-specific mechanistic support, cuproptosis remains at an early exploratory stage, and pyroptosis offers the most direct link to immune remodeling but with context-dependent and less predictable consequences. Their therapeutic value may lie in exposing subtype-specific vulnerabilities and enabling rational combination strategies, particularly with immunotherapy. Nevertheless, clinical translation remains limited by tumor heterogeneity, incomplete breast cancer-specific mechanistic evidence, the lack of robust biomarkers for patient selection, and the risk of oxidative, metabolic, or inflammatory toxicity. Moreover, many current conclusions still rely on preclinical studies or pan-cancer extrapolation. Future progress will require biomarker-guided stratification, more direct validation of immune effects in breast cancer, and careful integration with existing treatment modalities.

## 5 Mitochondrial control of immune and stromal compartments in the breast cancer microenvironment

### 5.1 Nutrient competition and metabolite-mediated immunosuppression in the TME

In breast cancer, metabolic immunosuppression arises not only from nutrient competition but also from the accumulation of signaling metabolites within the tumor microenvironment (TME) (16, 99). Tumor cells compete with immune cells for glucose, glutamine, and arginine, while simultaneously generating lactate and other metabolites that directly impair antitumor immunity (24, 100, 101). These pressures reduce mitochondrial fitness in CD8+ T cells and NK cells, limiting proliferation, cytokine production, and cytotoxicity (16, 100, 102). Lactate-rich, acidic conditions further suppress dendritic-cell function and reinforce immune exhaustion (100, 103, 104). Thus, immune dysfunction in the breast cancer TME reflects the combined effects of nutrient deprivation and metabolite-mediated signaling rather than competition alone (16, 99, 105).

**Figure 1:**
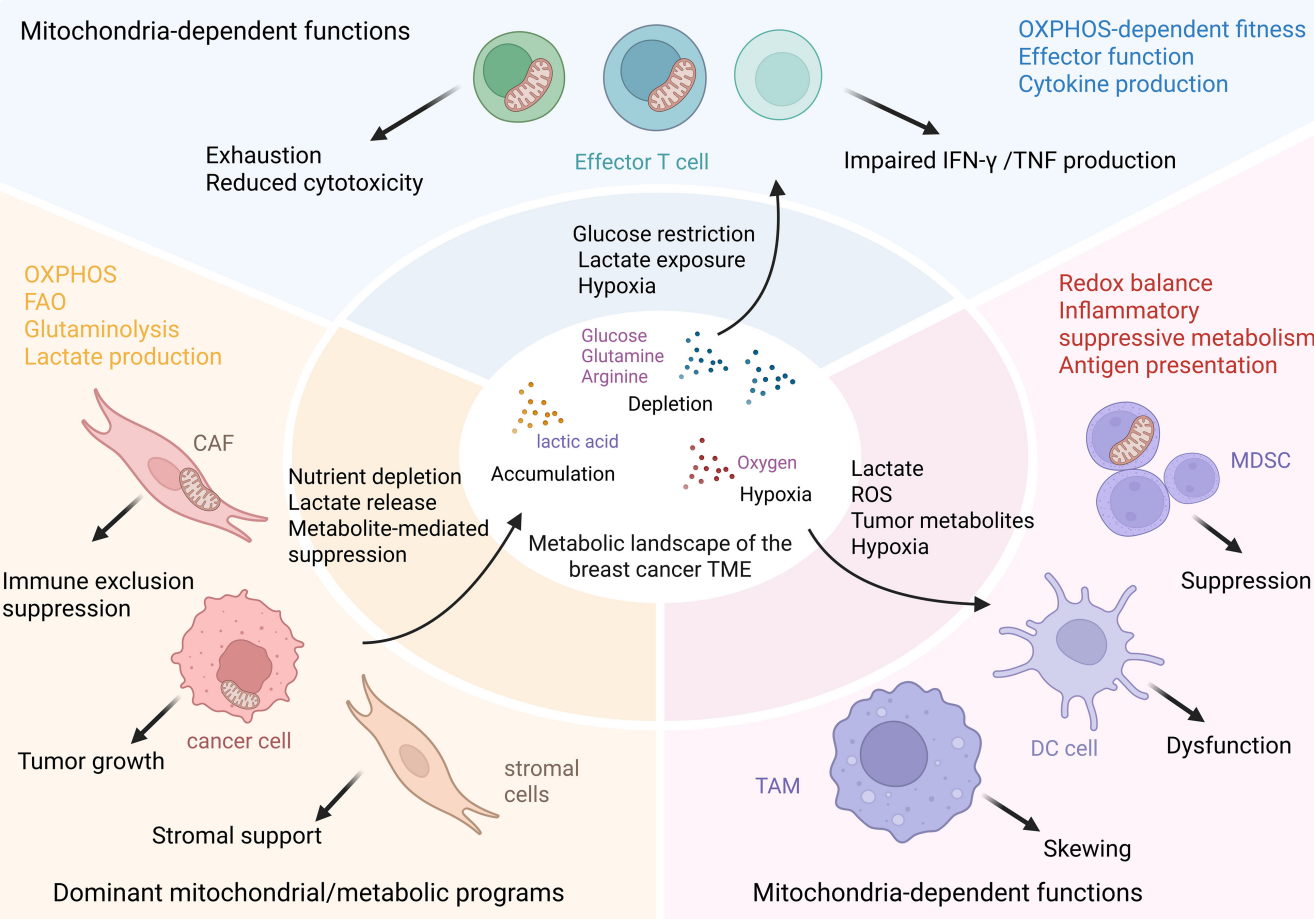
Metabolic competition and metabolite-mediated immune suppression in the breast cancer tumor microenvironment. Breast cancer cells, cancer-associated fibroblasts (CAFs), and other stromal cells actively shape a hostile metabolic landscape within the TME through high nutrient consumption and the release of suppressive metabolites. Their dominant metabolic programs—including oxidative phosphorylation (OXPHOS), fatty acid oxidation (FAO), glutaminolysis, and lactate-associated metabolic activity—promote glucose, glutamine, and arginine depletion, together with lactate accumulation, hypoxia, and acidic stress. These changes support tumor growth, reinforce stromal support, and contribute to immune exclusion and immunosuppression. By contrast, adaptive immune cells do not primarily generate this competitive environment, but instead are functionally constrained by it. Effector T cells rely on mitochondrial fitness and OXPHOS-dependent programs to sustain cytotoxicity and cytokine production; under conditions of glucose restriction, lactate exposure, and hypoxia, they undergo exhaustion, show reduced killing capacity, and display impaired IFN-γ and TNF production. Innate immune populations are also reshaped by this shared metabolic milieu, but their responses are manifested more as functional skewing than classical exhaustion. Lactate, reactive oxygen species (ROS), tumor-derived metabolites, and hypoxia alter redox balance, inflammatory programming, and antigen presentation, thereby promoting tumor-associated macrophage (TAM) polarization, myeloid-derived suppressor cell (MDSC)-mediated suppression, and dendritic cell (DC) dysfunction. Collectively, this figure highlights the breast cancer TME as a metabolically competitive ecosystem in which tumor and stromal compartments create the suppressive niche, while adaptive and innate immune cells exhibit distinct mitochondria-dependent forms of dysfunction. Therapeutically, these interactions support strategies aimed at disrupting tumor–stroma metabolic coupling, restoring immune-cell mitochondrial fitness, reprogramming myeloid metabolism, and improving responses to immune checkpoint blockade (ICB).

These metabolic interactions and their immunological consequences are schematically summarized in **Figure 1**.

### 5.2 mtDAMPs and innate immune sensing in breast cancer

Mitochondrial damage also shapes breast cancer immunity through the release of mtDAMPs, particularly mtDNA, ATP, and mitochondrial ROS (49, 106). Cytosolic mtDNA can activate cGAS-STING signaling and downstream type I interferon responses, thereby promoting dendritic-cell activation and T-cell priming, especially under therapy-induced stress (49, 107, 108). Extracellular ATP may engage purinergic receptors such as P2X7 and contribute to inflammasome activation in macrophages and other myeloid cells, while mitochondrial ROS can further amplify innate immune signaling and inflammatory cytokine production (109–112). Additional mitochondrial signals, including cardiolipin, mtRNA, and N-formyl peptides, may extend this danger-sensing repertoire, although their specific roles in breast cancer remain less clearly defined (106, 111, 113). In breast cancer, these pathways are particularly relevant in the context of chronic hypoxia, treatment-induced mitochondrial injury, and myeloid-rich immunosuppressive microenvironments (49, 107, 112, 114). Importantly, mtDAMP signaling is context dependent: acute mitochondrial stress may enhance immune recognition and antitumor priming, whereas persistent mtDAMP release may instead sustain maladaptive inflammation, myeloid skewing, immune dysfunction, and tumor progression (106, 111, 113).

**Figure 2:**
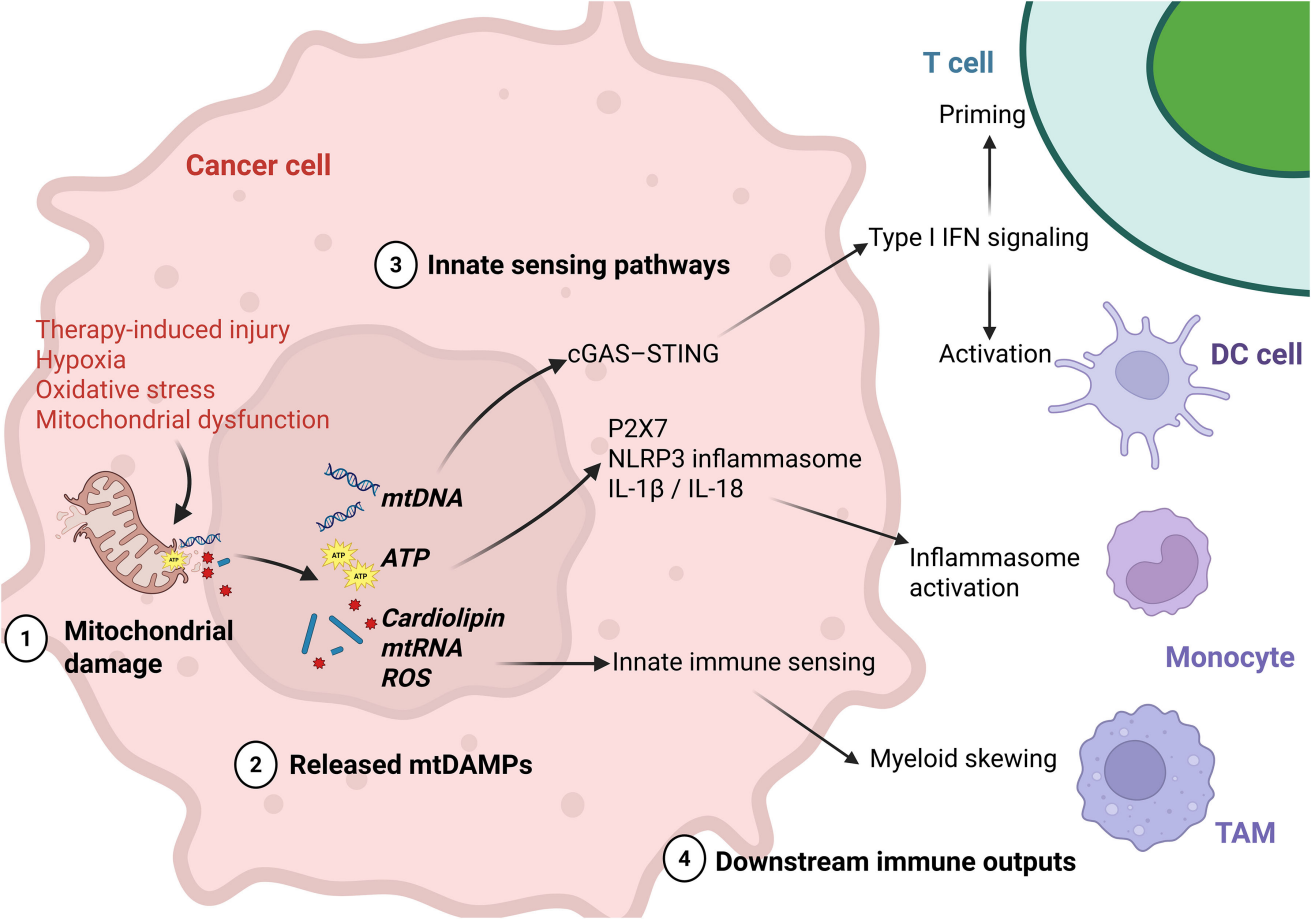
mtDAMP release and innate immune sensing in breast cancer. Mitochondrial stress in breast cancer cells can be triggered by therapy-induced injury, hypoxia, oxidative stress, and intrinsic mitochondrial dysfunction, leading to mitochondrial damage and the release or exposure of mitochondrial danger-associated molecular patterns (mtDAMPs). Among these signals, mitochondrial DNA (mtDNA), extracellular ATP, and other mitochondrial danger signals such as cardiolipin, mitochondrial RNA (mtRNA), and reactive oxygen species (ROS) serve as key initiators of innate immune sensing. Released mtDNA can activate the cGAS–STING pathway and promote type I interferon (IFN) signaling, thereby supporting dendritic-cell activation and T-cell priming. Extracellular ATP can engage P2X7 and stimulate NLRP3 inflammasome signaling, leading to IL-1β and IL-18 production and inflammasome activation. Cardiolipin, mtRNA, and ROS further amplify innate sensing through pattern-recognition and inflammasome-associated pathways, contributing to myeloid activation, inflammatory reinforcement, and functional skewing of innate immune cells. These signals collectively generate context-dependent downstream immune outputs. In acute or therapy-associated settings, mtDAMP signaling may enhance immune priming through type I IFN responses, dendritic-cell activation, and improved T-cell initiation. In contrast, persistent or excessive mtDAMP release may favor maladaptive inflammation, inflammasome-driven cytokine production, myeloid skewing, and chronic immune dysfunction. Overall, the figure illustrates how damaged mitochondria act as a source of innate immune signals in breast cancer, linking mitochondrial injury to both immune activation and immunopathologic inflammation in a context-dependent manner.

The principal mtDAMP sensing pathways and their downstream immune consequences are summarized in **Figure 2**.

### 5.3 Immune-cell and stromal mitochondrial fitness in breast cancer

Mitochondrial fitness is a key determinant of immune-cell performance in breast cancer (115). In T cells, and likely also in NK cells, chronic antigen stimulation, hypoxia, glucose restriction, glutamine limitation, and lactate accumulation can drive mitochondrial stress, ROS imbalance, and functional exhaustion, thereby weakening antitumor responses, although direct breast-cancer-specific evidence for NK-cell mitochondrial exhaustion remains more limited (116–119). Myeloid populations are similarly shaped by mitochondrial metabolism: macrophage polarization, MDSC-mediated suppression, and dendritic-cell dysfunction are all influenced by redox balance and metabolic rewiring, reinforcing an immunosuppressive TME (120–122).

Beyond immune cells, cancer-associated fibroblasts (CAFs) also undergo substantial mitochondrial and metabolic remodeling (123, 124). CAFs can enhance OXPHOS and produce lactate, ketone bodies, and other metabolites that fuel tumor growth and suppress antitumor immunity (116, 123, 125). CAF-derived metabolic signals may also promote mitochondrial biogenesis and therapy resistance in cancer cells (126–128). In breast cancer, however, the most defensible conclusion is that CAF-associated mitochondrial remodeling and metabolic coupling likely influence the immune microenvironment indirectly, whereas direct evidence linking canonical mitochondrial dynamics in CAFs to immune regulation remains limited (123, 129). Thus, stromal mitochondrial rewiring should be regarded as an important but still incompletely defined contributor to tumor progression and therapeutic resistance (124, 130).

Taken together, mitochondrial regulation of the breast cancer microenvironment extends beyond tumor cells alone and integrates nutrient stress, suppressive metabolites, innate danger sensing, immune-cell bioenergetics, and stromal metabolic coupling (115, 123). This broader framework helps explain how mitochondrial dysfunction shapes both tumor progression and treatment response (130).

These mitochondrial-linked processes represent important but unevenly developed therapeutic opportunities in breast cancer (115, 130). Their translational potential lies in identifying subtype-specific vulnerabilities and informing rational combinations with immunotherapy, targeted therapy, or chemotherapy (131). However, progress remains limited by tumor heterogeneity, incomplete breast cancer-specific mechanistic evidence, underdeveloped biomarkers, and the risk of oxidative, metabolic, or inflammatory toxicity. Future advances will therefore depend on biomarker-guided stratification, clearer validation of immune effects in breast cancer, and more precise targeting of mitochondrial pathways across both immune and stromal compartments.

## 6 Therapeutic opportunities and immune liabilities of mitochondrial targeting in breast cancer

### 6.1 Strategies with breast cancer and immune relevance

Mitochondria-targeted strategies in breast cancer are most compelling when they affect not only tumor bioenergetics but also the immune context of the tumor microenvironment. Among these, OXPHOS inhibition has attracted particular interest in metabolically plastic or therapy-resistant breast cancers, where OXPHOS-high states may represent actionable vulnerabilities (132). In tumor cells, complex I inhibition can induce energetic stress, suppress proliferation, and limit survival in OXPHOS-dependent settings. At the same time, selected preclinical studies suggest that OXPHOS inhibition may enhance the efficacy of immune checkpoint blockade by reshaping the TME although these effects remain highly context dependent (132). This therapeutic rationale, however, has not translated easily into clinical development. Early-phase clinical evaluation of the complex I inhibitor IACS-010759 was limited and ultimately discontinued because of dose-limiting toxicities, including blood lactate elevation and neurological adverse events, highlighting the narrow therapeutic window of systemic OXPHOS inhibition (133).

Representative mitochondria-targeted strategies with breast cancer and immune relevance are summarized in **Table 1**.

**Table 1:** Summary of representative mitochondria-related therapeutic and immunometabolic strategies under investigation in breast cancer.

Strategy	Target/mechanism	Evidence in breast cancer	Immune relevance	Development status	Key limitation/immune-cell liability
OXPHOS inhibition + ICB	Complex I/OXPHOS inhibition (e.g., IACS-010759-based rationale; metformin-related OXPHOS modulation) combined with checkpoint blockade	Preclinical BC models support OXPHOS dependence in resistant or metabolically plastic tumors	May improve CD8+ T-cell infiltration/function and sensitize selected tumors to ICB	Preclinical in BC; IACS-010759 clinical development limited/discontinued	Narrow therapeutic window; may also impair immune-cell mitochondrial function; metabolic compensation
Metformin-related immunomodulation	Mild mitochondrial complex I inhibition/AMPK-linked metabolic rewiring	Preclinical and translational evidence supports modulation of tumor metabolism and therapy sensitization in BC	May reduce hypoxia-associated immunosuppression and improve T-cell fitness in selected settings	Preclinical/translational; mixed clinical relevance	Context dependence; modest potency; limited predictive biomarkers
ATAD3A–PINK1–PD-L1 axis	Mitophagy-linked mitochondrial PD-L1 regulation	Demonstrated in TNBC, especially in chemoimmunotherapy resistance	Directly links mitochondrial quality control to immune escape and PD-1 blockade sensitivity	Preclinical	Subtype-restricted evidence; not yet clinically validated
STING agonist nanoparticle delivery	Delivery-enabled mitochondrial/innate immune activation via STING signaling	Preclinical evidence in immune-cold BC/TNBC models	Enhances dendritic-cell activation, type I IFN signaling, and T-cell priming	Preclinical	Delivery-dependent; inflammatory toxicity risk
Sonodynamic therapy (SDT) + ICB	Mitochondria-centered ROS generation and immunogenic stress combined with checkpoint blockade	Preclinical BC models show tumor killing and immune potentiation	May amplify antitumor immunity and enhance ICB efficacy	Preclinical	Selectivity, dosing, and reproducibility remain uncertain

Rows summarize the principal target or mechanism, current evidence in breast cancer, potential immune relevance, development status, and key translational limitations, with emphasis on mitochondrial metabolism, mitophagy-linked immune escape, innate immune activation, and combination strategies with immune checkpoint blockade.

Strategies listed are representative rather than exhaustive and are intended to highlight major mitochondria-centered or mitochondria-relevant therapeutic directions currently supported mainly by preclinical or translational evidence in breast cancer. “Development status” refers to the most advanced stage of evidence described for each strategy in the breast cancer setting. Because mitochondrial interventions may affect both tumor cells and immune cells, key limitations include not only tumor-intrinsic resistance and delivery barriers, but also potential immune-cell liability and context-dependent effects on antitumor immunity.

OXPHOS, oxidative phosphorylation; ICB, immune checkpoint blockade; AMPK, AMP-activated protein kinase; PD-L1, programmed death-ligand 1; PINK1, PTEN-induced kinase 1; STING, stimulator of interferon genes; IFN, interferon; SDT, sonodynamic therapy; ROS, reactive oxygen species; TNBC, triple-negative breast cancer.

Metformin provides a broader immunometabolic example. Beyond its effects on tumor mitochondrial metabolism, metformin has been reported to reduce hypoxia-associated immunosuppression, improve T-cell metabolic fitness, and enhance sensitivity to chemotherapy or immunotherapy in selected breast cancer models (134). These observations suggest that some mitochondrial modulators may exert dual effects on tumor cells and immune cells, rather than functioning solely as tumor-intrinsic metabolic inhibitors (135, 136).

More direct links between mitochondrial regulation and immune escape have emerged from studies of mitophagy and checkpoint control. In TNBC, the ATAD3A–PINK1–PD-L1 axis connects mitochondrial quality control to immune resistance: altered mitophagy can stabilize mitochondrial PD-L1 signaling and thereby limit response to PD-1 blockade (44, 137). This makes the pathway especially relevant because it links mitochondrial homeostasis in tumor cells to downstream immunotherapy sensitivity.

Additional approaches aim to activate innate or immunogenic signaling through mitochondrial stress. STING agonist delivery is of interest because it can enhance innate immune sensing and promote T-cell priming in otherwise poorly inflamed breast tumors, although its efficacy remains strongly dependent on delivery strategy and treatment context (138–140). Likewise, sonodynamic therapy (SDT) exploits mitochondria-centered ROS generation to induce immunogenic stress and has shown the potential to amplify checkpoint blockade responses in preclinical breast cancer models (141). These strategies are particularly attractive because they extend mitochondrial targeting beyond metabolic suppression alone and instead engage immune activation pathways. Additional evidence suggests that intercellular mitochondrial transfer within the tumor microenvironment may also contribute to immune evasion and therapeutic resistance, thereby supporting mitochondrial transfer as a potential therapeutic target in breast cancer (142).

By contrast, BH3 mimetics remain mechanistically attractive but less straightforward in clinical translation. While they can restore mitochondrial apoptosis in anti-apoptotic tumor states, their benefit in breast cancer appears more dependent on subtype, biomarker selection, and adaptive survival circuitry than initially expected. For this reason, they are better interpreted as a context-dependent component of mitochondrial targeting rather than a universally effective breast cancer strategy (44).

Overall, the most relevant mitochondria-directed strategies in breast cancer are those that combine tumor metabolic vulnerability with measurable immune consequences. This includes OXPHOS inhibition plus checkpoint blockade, metformin-related immunomodulation, mitophagy-checkpoint regulation, STING-based delivery systems, and sonodynamic approaches. However, their effects are not cell-type neutral, and future development will require more explicit evaluation of how these agents influence both tumor cells and antitumor immune populations.

### 6.2 From mechanistic promise to translational uncertainty

Despite strong mechanistic rationale, the clinical translation of mitochondrial targeting in breast cancer remains more challenging than preclinical studies initially suggested. A central reason is that mitochondrial functions exploited by tumor cells are not unique to tumor cells. The same processes targeted to suppress tumor growth—such as OXPHOS, redox homeostasis, mitophagy, and mitochondrial apoptosis regulation—are also essential for immune-cell activation, persistence, and effector function. T cells, in particular, require intact mitochondrial fitness and oxidative metabolism to sustain proliferation, cytokine production, and long-term antitumor activity under chronic stimulation (143). Accordingly, systemic mitochondrial inhibition may generate a therapeutic paradox: while impairing tumor bioenergetics, it may simultaneously compromise antitumor immunity (144).

This concern is especially relevant for strategies targeting OXPHOS or mitochondrial respiration. Although OXPHOS inhibition may expose vulnerabilities in metabolically plastic or therapy-resistant breast tumors, its broader effects are unlikely to be cell-type neutral (69, 133). Cytotoxic lymphocytes and other immune populations also depend on mitochondrial metabolism to function within the nutrient-deprived and hypoxic tumor microenvironment. Thus, systemic mitochondrial inhibition may weaken tumor cells and antitumor immune cells at the same time, and the net effect may vary substantially depending on dose, timing, tumor subtype, and the metabolic state of infiltrating immune cells. These considerations help explain why impressive mechanistic promise does not necessarily translate into broad clinical benefit (69).

Tumor heterogeneity and adaptive metabolic rewiring further complicate this landscape (145). Breast cancers differ markedly in OXPHOS dependence, antioxidant capacity, mitophagy activity, and anti-apoptotic signaling, meaning that a given mitochondria-directed therapy is unlikely to be uniformly effective across subtypes or treatment settings. Even when an initial mitochondrial vulnerability exists, tumor cells may compensate through glycolytic switching, altered substrate utilization, or rewiring toward alternative survival pathways. Similar principles apply to mitochondrial apoptosis targeting. Although BH3 mimetics offer a mechanistically attractive means of restoring apoptosis, their clinical translation in breast cancer has been less straightforward than initially anticipated. For example, the overall negative result of the VERONICA trial suggests that BCL-2 dependence alone is insufficient to predict benefit in unselected ER-positive disease. Instead, tumor heterogeneity, adaptive survival signaling, and inadequate biomarker selection remain major barriers to effective translation (69).

Toxicity is another major limitation. Because mitochondrial pathways are fundamental to normal tissue homeostasis, strategies that disrupt respiration, redox balance, or mitochondrial quality control may carry substantial systemic liabilities (133). This issue is particularly important in highly oxidative tissues and in immune cells, where unintended mitochondrial injury may narrow the therapeutic window. In parallel, strategies designed to amplify mitochondrial stress or inflammatory signaling may also provoke oxidative, metabolic, or immune-related toxicity if not adequately controlled. Therefore, the challenge is not simply to identify mitochondria as a target, but to determine when, where, and in which cellular compartment mitochondrial intervention is therapeutically advantageous.

Taken together, these limitations indicate that mitochondrial targeting in breast cancer should not be presented as a uniformly promising platform, but rather as a context-dependent strategy that requires careful refinement. Future progress will depend on better biomarkers of mitochondrial dependence, clearer distinction between tumor-selective and immune-cell liabilities, and rational combination approaches that minimize collateral impairment of antitumor immunity (144, 145).

### 6.3 Tumor-selective delivery and future therapeutic design

These challenges make tumor-selective delivery a central requirement for the future development of mitochondria-targeted therapies in breast cancer (146). Because many mitochondrial processes are shared by tumor cells, immune cells, and normal oxidative tissues, systemic exposure may narrow the therapeutic window and undermine antitumor immunity. Accordingly, delivery systems are not merely technical refinements but may determine whether mitochondrial interventions can achieve meaningful selectivity *in vivo* (147).

Several strategies may help address this problem. Nanoparticles, receptor-targeted carriers, exosome-based platforms, and pH- or hypoxia-responsive systems offer the potential to enrich mitochondrial drugs within tumors while limiting off-target exposure (147–150). Such approaches are particularly relevant for agents that disrupt respiration, amplify ROS, or activate innate immune pathways, where uncontrolled systemic distribution may produce metabolic or inflammatory toxicity (151). In breast cancer, these platforms may also help tailor mitochondrial therapies to subtype-specific contexts, such as hypoxic, OXPHOS-dependent, or immune-cold tumors.

Future therapeutic design should therefore move beyond broad mitochondrial inhibition toward more precise, compartment-aware intervention. This will require integrating biomarkers of mitochondrial dependence, mitophagy activity, redox state, or anti-apoptotic rewiring with rational combination strategies and delivery systems that preferentially target tumor or stromal compartments while sparing antitumor immune cells. In this sense, the next phase of mitochondrial therapy in breast cancer will depend less on identifying new mitochondrial targets alone than on improving selectivity, biological stratification, and immune compatibility (146, 152, 153).

## 7 Challenges, biomarkers, and future directions

### 7.1 Current barriers to clinical translation

The clinical translation of mitochondria-targeted strategies in breast cancer remains limited by several interconnected challenges. First, mitochondrial dependencies vary substantially across breast cancer subtypes, treatment states, and microenvironmental contexts, while tumor cells can adapt under therapeutic pressure through metabolic plasticity and alternative substrate use (154, 155). As a result, mitochondrial vulnerabilities are often context dependent rather than broadly actionable.

Second, mitochondrial targeting raises important safety and specificity concerns. Because pathways such as OXPHOS, redox homeostasis, mitophagy, and apoptotic regulation are shared by tumor cells, immune cells, and normal oxidative tissues, systemic interventions may narrow the therapeutic window and unintentionally impair antitumor immunity. This is particularly relevant for T cells, which require mitochondrial fitness and oxidative metabolism to sustain activation and effector function (133, 154). Finally, predictive biomarkers remain underdeveloped. Although OXPHOS-high states, mitophagy-related pathways, anti-apoptotic rewiring, and mitochondrial stress signatures are all promising candidates, few have been robustly validated for patient selection in breast cancer (156–158).

### 7.2 Biomarker-guided and immune-aware future directions

Future progress is most likely to come from strategies that are both biomarker-guided and immune-aware. In the near term, the most feasible advances include improving tumor-selective delivery and refining patient stratification. Receptor-targeted nanoparticles, pH- or hypoxia-responsive carriers, and exosome-based systems may enhance intratumoral drug accumulation while reducing exposure of immune cells and normal tissues (147, 149, 159). At the same time, future studies should prioritize biomarkers that capture mitochondrial dependence, mitophagy activity, redox imbalance, and anti-apoptotic rewiring across both tumor and stromal compartments (158, 160).

More speculative directions, including AI-guided drug discovery, mitochondrial gene editing, and engineered mitochondrial replacement, remain scientifically interesting but are less likely to shape near-term breast cancer practice (161, 162). Overall, the future of mitochondrial targeting in breast cancer will depend less on identifying new mitochondrial targets alone than on improving selectivity, biological stratification, and immune compatibility.

## 8 Conclusion

Mitochondria have emerged as central regulators of breast cancer biology, integrating metabolic plasticity, redox balance, regulated cell death, and immune signaling across tumor, immune, and stromal compartments. Their dysregulation not only supports tumor growth and therapy resistance but also reshapes the tumor microenvironment through metabolic competition, mtDAMP signaling, immune-cell dysfunction, and stromal coupling. These context-dependent roles help explain why mitochondrial pathways represent compelling yet unevenly developed therapeutic opportunities in breast cancer.

Accordingly, the future of mitochondrial targeting will depend less on broad inhibition of mitochondrial function than on biomarker-guided, immune-aware, and tumor-selective intervention. Progress will require clearer definition of mitochondrial dependencies across breast cancer subtypes, improved distinction between tumor vulnerabilities and immune-cell liabilities, and more precise delivery strategies that enhance selectivity while limiting toxicity. In this sense, mitochondria should be viewed not simply as metabolic organelles, but as actionable integrators of tumor progression, immune regulation, and therapeutic response in breast cancer.
